# Studies over the existence of a certain impulse-based fuzzy integrodifferential equations of the Sobolev type

**DOI:** 10.1186/s13104-023-06638-y

**Published:** 2024-01-29

**Authors:** B. Radhakrishnan, M. Nagarajan, P. Anukokila, P. Shanmugasundram

**Affiliations:** 1 Department of Mathematics, PSG College of Technology, Coimbatore-04, TamilNadu India; 2 Department of Mathematics, Sri Sai Ranganathan Engineering College, Coimbatore, TamilNadu India; 3 Department of Mathematics, PSG College of Arts & Science, Coimbatore-14, TamilNadu India; 4 Department of Mathematics and Computational Sciences, Mizan-Tepi University, Tepi, Ethiopia

**Keywords:** Existence and uniqueness, Fuzzy solution, Neutral integrodifferential equation, Impulsive differential equation, Fixed point theorem, 34A07, 34A12, 12H20, 47H20, 34K45

## Abstract

The present investigation employs impulses and a non-local constraint to prove the existence are some various types of abstract differential and integrodifferential equations related to the Sobolev type. Semigroup theory, specifically variants of constant formula, is utilized to get the analytical results for those equations. Furthermore, findings using the Banach fixed point approach were examined using fuzzy numbers with values spanning the $\mathscr {E}_n$ range, which includes the normal, convex, upper semi-continuous, and compactly supported interval. A description is given for each situation to illustrate the principle.

## Introduction

There are numerous applications for the intriguing theory of differential equations throughout abstract spaces in the fields of analysis and other mathematics. Ordinary differential equations (ODEs), functional differential equations, partial differential equations (PDEs), and sometimes a combination of interacting systems of ordinary and partial differential equations would be used, depending on the nature of the problems. In the fields of applied mathematics, engineering, biology, and the physical sciences, nonlinear differential and integral equations in abstract spaces have been utilized to deal with several problems. With significant applications in many areas of analysis and other disciplines, the theory of nonlinear differential and integral equations in abstract spaces is growing quickly.

The fuzzy semigroups of linear operators to solve fuzzy differential equations were originally proposed in the fuzzy literature by the authors of Gomes et al. [1]. Next, fuzzy Cauchy problems were studied by Kaleva [2] using nonlinear iteration semigroups (with exponential formula). Ding and Kandel [3] examined how differential equations and fuzzy sets may be used to create fuzzy logic systems, also known as fuzzy dynamical systems, which are similar to fuzzy neutral functional differential equations. With its wide range of applications, semigroup theory has recently been the subject of much study in the classic literature. For the interested reader, we recommend the fascinating work by Pazy [4], in which the author uses semigroups to solve partial and ordinary differential equations.

PDEs are frequently employed to simulate a broad variety of scientific and engineering issues. They are typically expressed in several forms of differential or integrodifferential equations in abstract spaces. Several writers have studied integrodifferential equations in abstract spaces, including [5–8]. In general, an integrodifferential equation is an abstract representation of a large number of partial integrodifferential equations that emerge in issues involving wave propagation and other physical phenomena. By addressing Sobolev type nonlinear interodifferential equations, Radhakrishnan et al. [9] investigated the existence of Sobolev type nonlinear neutral integrodifferential equations. Many writers have analyzed differential equations of the Sobolev type [10–12].

Nonlocal Cauchy problem, namely, the differential equation with a non-local initial condition $z(\tau _0) + e(\tau _1,\ldots ,\tau _n, z) = z_0$ $(0\le \tau _0< \tau _1<\ldots < \tau _n\le \tau _0 + a$ and *e* is a given function) is one of the important topics in the study of analysis. The primary motivation behind interest in the area revolves around the non-local initial condition’s high efficacy over the standard one while treating physical issues. In fact, the conventional beginning condition $z(0) = z_0$ could not be incorporated into a number of fascinating empirical events that the non-local initial condition represents. For instance, the function $e(\tau _1,\ldots ,\tau _n, z)$ may be given by

$$(\tau _1,\ldots ,\tau _n, z) =\displaystyle \sum _{k=1}^{n}c_k z(\tau _k)$$

$(c_k,\ k = 1,\ldots ,n$ are constants). In this case, we are permitted to have the measurements at $\tau = 0, \tau _1,\ \ldots ,\tau _n,$ rather than just at $\tau = 0.$ More specially, letting $e(\tau _1,\ldots , \tau _n, z) = -z(\tau _k)$ and $z_0 = 0$ yields a periodic problem and letting $e(\tau _1,\ldots , \tau _n, z) = -z(\tau _0) + z(\tau _n)$ gives a backward problem. Byszewski [13] was the first to delve at the existence of solutions to evolution equations in Banach spaces with non-local constraints.

In reality, experts agree that discretely emerging discontinuities enrich its continuous. The latter are also known as jumps or, from an energy standpoint, impulses. Many evolution processes are accompanied by abrupt shifts in condition at specific points in time. These processes are subject to short-term perturbations that are insignificant in contrast to the process’s lifespan. As a result, it’s reasonable to presume that these disturbances occur instantly, in the form of impulses. As a result, differential equations with impulsive effects serve as a natural description of observable evolution events in a variety of real-world issues, such as [14–21]. In some situations, such as the so-called neutral differential difference equations, the delayed argument occurs in both the derivative of the state variable and the independent variable. A neutral functional differential equation involves the derivatives concerning past events history or derivatives of functionals of the prior history, and the present state of the system. The book on neutral functional differential equations by Hale and Verduyn Lunel [22] and its references are a useful resource.

In accordance with the premise that “all things occurring in the real world are unstable and unexpected,” Zadeh[23] developed fuzzy set theory in 1965. In a number of research areas, the notion was proposed and implemented successfully. This hypothesis has lately been investigated further, with a variety of applications being proposed. To explain fuzzy conceptions, Diamand et al. [24] established the metric space of fuzzy sets theory. Kaleva [25, 26] looked at fuzzy differential equations in broad sense. For further discussion on the several types of fuzzy differential equations, see [27–32].

Motivated by the literature, we are using the fixed point approach to investigate several types of Sobolev type Fuzzy neutral integro-differential equations with impulses in a fuzzy environment.

## Problem formulation

The authors of this paper have to investigate if fuzzy neutral impulsive nonlinear integrodifferential equations of the Sobolev type are subject to non-local conditions

1.1 $$\begin{aligned}{}[\mathbb {B}z(\nu )+ \mathscr {P}(\tau , z(\nu ))]^\prime +\mathbb {A}z(\nu )= \,& {} \mathbb {F}(\nu ,z(\nu ))+\int _0^\nu {\mathscr {K}}\Big (\nu ,\mu , z(\mu )\Big )d\mu \nonumber \\{} & {} \; \hspace{3cm} \nu \in [0,a], \nu \ne \nu _k \end{aligned}$$

1.2 $$\begin{aligned} z(0)+\sum _{i=1}^n c_i z(\nu _i)= & {} z_0 \end{aligned}$$

1.3 $$\begin{aligned} \Delta z(\nu _k)= & {} {\mathcal {I}}_k(z_{\nu _k}),\ \ k=1,2,...,m, \end{aligned}$$

where $\mathbb {A,B}:\mathbb J\rightarrow \mathbb {E}_n$ denotes a fuzzy coefficient and $\mathbb J=[0,a]$. The set of all upper semi continuous convex regular numbers that are uncertain, with limited $\lambda -$level intervals is designated as $\mathbb {E}_n$. The function $\mathbb F, {\mathscr {P}}, \mathscr {K}: \mathbb J\times \mathbb {E}_n\rightarrow \mathbb {E}_n$ is nonlinear fuzzy function and $z_0$ is a initial value and ${\mathcal {I}}_k z(\nu _k)=\Delta z(\nu _k)=z(\nu _k^+)-z(\nu _k^-),$ for all $k=1,2,...,m;\ 0 = \nu _0< \nu _1< \nu _2<...< \nu _m < \nu _{m+1} = a;$

## Preliminaries

By giving each $z \in \mathbb { R}^n$ a membership grade, a fuzzy subset of $\mathbb { R}^n$ is constructed using a membership function. In this way, the purpose of membership is addressed.

$$\begin{aligned} u: \mathbb R^n\ \text{ to } \text{ the } \text{ closed } \text{ interval }\ [0,1]. \end{aligned}$$

Across the analysis, the subsequent requirements were introduced: *w* maps $\mathbb {R}^n$ onto [0, 1], $[u]^0$ constitutes a finite subset of $\mathbb {R}^n$, *u* is fuzzy convex, and *u* is upper semi-continuous. In this instance, consider $\mathbb {E}_n$ represent the space of all fuzzy subsets *u* of $\mathbb {R}_n$, encompassing upper semi-continuous, normal, and fuzzy convex sets along with bounded supports. The space of all fuzzy subsets *u* of $\mathbb R$ is precisely expressed by $\mathbb E^1$.

A fuzzy amount $\mathscr {A}$ in $\mathbb R$ comprises a set that is fuzzy defined by the membership function $\chi _A$ spanning $\mathbb R\ \text{ to }\ [0, 1].$ A number that is fuzzy $\mathscr {A}$ also transforms into

$$\begin{aligned} \mathscr {A} = \int _{z\in \mathbb R} \frac{\chi _{\mathscr {A}}}{z} \end{aligned}$$

with $\chi _{\mathscr {A}}(\cdot )$ in the closed interval 0 and 1

Let *z* in $\mathbb R^n$ and $\mathcal D$ be a nonempty subsets of $\mathbb R^n$. Now the Hausdroff separation of $\mathcal B$ from $\mathcal D$ is defined by

$$\begin{aligned} d(z, \mathcal D)=\inf \{\left\| z-b\right\| : b\in \mathcal D\}. \end{aligned}$$

Let $\mathcal D$ and $\mathcal B$ be nonempty subsets of $\mathbb R^n$. The Hausdroff separation of $\mathcal B$ from $\mathcal D$ is defined by

$$\mathscr {H}_*^d (\mathcal B,\mathcal D)=\sup \{d(b, \mathcal D):b\in \mathcal B \}.$$

In general,

$$\mathscr {H}_*^d( \mathcal B,\mathcal D)\ne \mathscr {H}_*^d(\mathcal D,\mathcal B).$$

With regard to two mathematical functions $\mathcal A$ and $\mathcal B$, the Hausdroff gap between them can be expressed as $\mathscr {H}^d(\mathcal A, \mathcal B)$. The greatest measure $d^\infty$ on $\mathbb {E}_n$ is characterized as

$$\begin{aligned} d^\infty (x,y)=\sup \{\mathscr {H}^d\Big ([x]^\lambda , [y]^\lambda \Big ): \lambda \in (0,1]\},\ \text{ for } \text{ all }\ x,\ y\in \mathbb {E}_n, \end{aligned}$$

and is obviously metric on $\mathbb {E}_n$.

The writers of this paper make an inference that there is an operator $\mathcal {E}$ on $\mathbb {E}_n$, which is provided by formula

$$\begin{aligned} \mathcal {E}= \; \Big [ {\mathcal {I}}+ \sum _{i=n}^n c_i \mathbb {B}^{-1} {\mathscr {S}}(\tau _i)\mathbb {B} \Big ]^{-1}. \end{aligned}$$

We primary investigate the following fuzzy functional differential equation (FFDE) along with non-local initial condition of Sobolev type

2.1 $$\begin{aligned} (\mathbb {B}z(\nu ))^\prime\,= \; & {} \mathbb {A} z(\nu ) +\mathbb {F}(\nu , z(\nu )),\ \ \ \nu \in \mathbb J=[0,a] \end{aligned}$$

2.2 $$\begin{aligned} z(0)+ & {} \sum _{i=1}^n c_i z(\nu _i)= z_0, \end{aligned}$$

where $\mathbb {A,B}:\mathbb {J}\rightarrow \mathbb {E}_n$ denotes a fuzzy coefficient and $\mathbb J=[0,a]$, $\mathbb {E}_n$ is the collection of all upper semi continuous convex normal fuzzy numbers with bounded $\lambda -$level intervals. The function $\mathbb {F}: \mathbb {J} \times \mathbb {E}_n\rightarrow \mathbb {E}_n$ is nonlinear fuzzy function and $z_0$ is a initial value.

### Definition 2.1

A continuous function $z(\nu )$ of the integral equation

$$\begin{aligned} z(\nu )\,= \; & {} \mathbb {B}^{-1}{\mathscr {S}}(\tau ) \mathbb {B}\mathcal {E} z_0 -\sum _{i=1}^n c_i\mathbb {B}^{-1} {\mathscr {S}}(\nu )\mathcal {E} \times \Big \{ \int _0^{\nu _i}\mathbb {B}^{-1}{\mathscr {S}}(\nu _i-\mu ) \mathbb {F}(\mu , z(\mu ))d\mu \Big \}\\+ & {} \int _0^\nu \mathbb {B}^{-1} {\mathscr {S}}(\nu -\mu ) \mathbb {F}(\mu , z(\mu ))d\mu + \mathbb {B}^{-1}\mathbb {A}{\mathscr {S}}(\nu ) \mathbb {B} \mathcal {E} z_0 \end{aligned}$$

is said be a solution, of problem (2.1, 2.2) on $\mathbb J$.

### Remark 2.1

A solution of (2.1, 2.2) satisfies the condition (2.2). Then

$$\begin{aligned} z(0)\,=\; & {} \mathcal {E}z_0+\mathbb {A}\mathcal {E} z_0 -\sum _{i=1}^n c_i\mathcal {E}\mathbb {B}^{-1} \Big \{ \int _0^{\nu _i}{\mathscr {S}}(\nu _i-\mu )\mathbb {B}^{-1}\mathbb {F}(\mu , z(\mu ))d\mu \Big \} \end{aligned}$$

and

$$\begin{aligned} z(\nu _j)\,= \; & {} \mathbb {B}^{-1}{\mathscr {S}}(\nu _j) \mathbb {B}\mathcal {E} z_0 -\sum _{i=1}^n c_i\mathbb {B}^{-1} {\mathscr {S}}(\nu _j)\mathbb {B}\mathcal {E} \Big \{ \int _0^{\nu _i}\mathbb {B}^{-1}{\mathscr {S}}(\nu _i-\mu ) \mathbb {F}(\mu , z(\mu ))d\mu \Big \}\\{} & {} +\int _0^\nu \mathbb {B}^{-1}\mathscr {S}(\nu _j-\mu )\mathbb {F}(\mu , z_\mu (\mu ))d\mu + \mathbb {B}^{-1}\mathbb {A}{\mathscr {S}}(\nu _j) \mathbb {B} \mathcal {E} z_0. \end{aligned}$$

Therefore

$$\begin{aligned} z(0)+\sum _{j=1}^n c_j z(\nu _j)= & {} \Big [ \mathscr {I} +\sum _{i=1}^n c_i z(\nu _i)\mathbb {B}^{-1} {\mathscr {S}}(\tau _j)\mathbb {B}\Big ]\mathcal {E}z_0-\Big [ \mathscr {I} +\sum _{i=1}^n c_i z(\nu _i)\mathbb {B}^{-1} {\mathscr {S}}(\tau _j)\mathbb {B}\Big ]\\\times & {} \sum _{i=1}^n c_i \mathcal {E} \Big \{ \int _0^{\nu _i} {\mathscr {S}}(\nu _i-\mu )\mathbb {B}^{-1} \mathbb {F}(\mu ,z(\mu ))d\mu \Big \}\\{} & {} +\int _0^{\nu _j} {\mathscr {S}}(\nu _j-\mu )\mathbb {B}^{-1} \mathbb {F}(\mu ,x_\mu (\mu ))d\mu = z_0. \end{aligned}$$

The subsequent assumptions are required in order to demonstrate the existence result:

($$A_1$$)
$\mathbb {A}: \mathbb {J}\rightarrow \mathbb {E}_n$ is a fuzzy coefficient and $\{\mathscr {C}(\nu ), \nu \in \mathbb {E}_n\}$ of bounded linear operator in Banach space. There exist constants $\mathbb {M}_s\ge 0, \mathbb {M}_c\ge 0$ such that $|{\mathscr {S}}(\nu )|\le \mathbb {M}_s$, for every $\nu \in [0,a]$. Furthermore, take $\mathbb {M}_a=\displaystyle \sup _{0\le t \le a}|\mathbb {A}{\mathscr {S}}(\nu )|$ and $\mathbb {M}_B=|\mathbb {B}^{-1}|$. Let $\mathbb {M}_c=\sum |c_i|$.
($$A_2$$)
The function $\mathbb {F}: \mathbb {J} \times \mathbb {E}_n \rightarrow \mathbb {E}_n$ satisfies the following conditions: $\mathscr {H}^d\Big ([\mathbb {F}(\nu , z(\nu )]^\lambda ,\mathbb {F}(\nu , y(\nu )]^\lambda \Big ) \le {\mathbb {L}}_f \mathscr {H}^d (z_\nu (\nu ),z_\nu (\nu ))$, for $\nu \in \mathbb {J}$ and $z_\nu , y_\nu \in \mathbb {E}_n$.
($$A_3$$)
For our convenience, we choose
  $$\begin{aligned}{} & {} \mathbb {M}_b\mathbb {M}_S|\mathbb {B}\mathcal {E}x_0| +b\mathbb {M}_b\mathbb {M}_s{\mathbb {L}}_fr+{\mathbb {L}} _c\mathbb {M}_b^2|{\mathscr {B}}\mathcal {E}|\mathbb {M}_cb \mathbb {M}_s{\mathbb {L}}_f\le \mathcal {L};\ \ \ \text{ and }\\ \delta= \; & {} b\mathbb {M}_b\mathbb {M}_s {\mathbb {L}}_f+b\mathbb {M}_c\mathbb {M}_b^2|\mathscr{B}\mathscr{E}| \mathbb {M}_c\mathbb {M}_s{\mathbb {L}}_f. \end{aligned}$$

## Existence and uniqueness

### Theorem 3.1

Assuming $(A_1)-(A_3)$ retain, there exists a solution for (2.1)-(2.2) on $\mathbb {J}$.

### Proof

Consider the subset $\mathcal {X}$ of $\mathbb {C}(\mathbb {J}, \mathbb {E}_n)$. specified through

$$\begin{aligned} \mathcal {X}=\{ z: z(\nu )\in \mathbb {C}(\mathbb {J},\ \mathbb {E}_n,\ |z(\nu )|\le r, \nu \in \mathbb {J}\}. \end{aligned}$$

Again, we define a mapping $\mathscr {F}:\mathcal {X} \rightarrow \mathcal {X}$ by

$$\begin{aligned} \mathscr {F} (z(\nu ))\,= \; & {} \mathbb {B}^{-1}\mathscr {C}(\nu ) \mathbb {B}\mathcal {E} z_0 -\sum _{i=1}^n c_i\mathbb {B}^{-1} \mathscr {C}(\nu )\mathcal {E} \Big \{ \int _0^{\nu _i}\mathbb {B}^{-1}{\mathscr {S}}(\nu _i-\mu ) \mathbb {F}(\mu , z_\mu (\mu ))d\mu \Big \}\\+ & {} \int _0^\nu \mathbb {B}^{-1} {\mathscr {S}}(\nu -\mu ) \mathbb {F}(\mu , z_\mu (\mu ))d\mu + \mathbb {B}^{-1}\mathbb {A}{\mathscr {S}}(\nu ) \mathbb {B} \mathcal {E} z_0. \end{aligned}$$

First to show that the operator $\mathscr {F}$ maps $\mathcal {X}$ into itself. Now

$$\begin{aligned} |\mathscr {F} (z(\nu ))|\le & {} |\mathbb {B}^{-1}\mathscr {C}(\nu )\mathbb {B}\mathcal {E}z_0|+|\int _0^\nu {\mathscr {S}}(\nu -\mu ) \mathbb {B}^{-1} \mathbb {F}(\mu , z(\mu ))d\mu |\\+ & {} |\sum _{i=1}^n c_i \mathbb {B}^{-1} \mathscr {C}(\nu )\mathbb {B}\mathcal {E}\int _0^{\nu _i}\mathbb {B}^{-1}{\mathscr {S}}(\nu _i-\mu ) \mathbb {F}(\mu ,z(\mu )) d\mu |\\\le & {} \mathbb {M}_b\mathbb {M}_c|{\mathscr {B}}\mathscr {E}x_0|+b\mathbb {M}_b\mathbb {M}_s{\mathbb {L}}_fr+{\mathbb {L}}_c\mathbb {M}_b^2|{\mathscr {B}}\mathscr {E}|\mathbb {M}_cb\mathbb {M}_s{\mathbb {L}}_f. \end{aligned}$$

From the assumption $(A_3)$, $|\mathscr {F} (z(\nu ))|\le \mathcal {L}$. Therefore the $\mathscr {F}$ maps $\mathcal {X}$ into itself. Also, if $x,y\in \mathcal {X}$.

$$\begin{aligned}{} & {} {{\mathcal {H}}^d\Big (\mathscr {F} (z(\nu ), \mathscr {F} (y(\nu )\Big )}\\= & {} \mathscr {H}^d\Big (\Big [\mathbb {B}^{-1}{\mathscr {S}}(\nu ) \mathbb {B}\mathcal {E} z_0 -\sum _{i=1}^n c_i\mathbb {B}^{-1} {\mathscr {S}}(\nu )\mathcal {E} \times \Big \{ \int _0^{\nu _i}\mathbb {B}^{-1}{\mathscr {S}}(\nu _i-\mu ) \mathbb {F}(\mu , z(\mu ))d\mu \Big \}\\{} & {} +\int _0^\nu \mathbb {B}^{-1} {\mathscr {S}}(\nu -\mu ) \mathbb {F}(\mu , z_\mu (\mu ))d\mu + \mathbb {B}^{-1}\mathbb {A}{\mathscr {S}}(\nu ) \mathbb {B} \mathcal {E} z_0\Big ]^\lambda ,\Big [\mathbb {B}^{-1}{\mathscr {S}}(\nu ) \mathbb {B}\mathcal {E} y_0 \\{} & {} -\sum _{i=1}^n c_i\mathbb {B}^{-1} {\mathscr {S}}(\nu )\mathcal {E} \times \Big \{ \int _0^{\nu _i}\mathbb {B}^{-1}{\mathscr {S}}(\nu _i-\mu ) \mathbb {F}(\mu , y(\mu ))d\mu \Big \}\\{} & {} +\int _0^\tau \mathbb {B}^{-1} {\mathscr {S}}(\nu -\mu ) \mathbb {F}(\mu , y(\mu ))d\mu + \mathbb {B}^{-1}\mathbb {A}{\mathscr {S}}(\nu ) \mathbb {B} \mathcal {E} y_0\Big ]^\lambda \Big )\\\le & {} \mathscr {H}^d\Big (\Big [\sum _{i=1}^n c_i\mathbb {B}^{-1} {\mathscr {S}}(\nu )\mathcal {E} \times \Big \{ \int _0^{\nu _i}\mathbb {B}^{-1}{\mathscr {S}}(\nu _i-\mu ) \mathbb {F}(\mu , z_\mu (\mu ))d\mu \Big \}\\{} & {} +\int _0^\nu \mathbb {B}^{-1} {\mathscr {S}}(\nu -\mu ) \mathbb {F}(\mu , z(\mu ))d\mu + \mathbb {B}^{-1}\mathbb {A}{\mathscr {S}}(\nu ) \mathbb {B} \mathcal {E} z_0\Big ]^\lambda ,\Big [\sum _{i=1}^n c_i\mathbb {B}^{-1}\mathscr {C}(\nu )\mathcal {E}\\{} & {} \times \Big \{ \int _0^{\nu _i}\mathbb {B}^{-1}{\mathscr {S}}(\nu _i-\mu ) \mathbb {F}(\mu , y(\mu ))d\mu \Big \}+\int _0^\nu \mathbb {B}^{-1} {\mathscr {S}}(\nu -\mu ) \mathbb {F}(\mu , y(\mu ))d\mu \Big ]^\lambda \Big )\\\le & {} [b\mathbb {M}_b\mathbb {M}_s {\mathbb {L}}_f+b{\mathbb {L}}_c\mathbb {M}_b^2|\mathbb {B}\mathcal {E}\mathbb {M}_c\mathbb {M}_s {\mathbb {L}}_f|\mathscr {H}^d\Big (z(\nu ), y(\nu )\Big ). \end{aligned}$$

Therefore,

$$\begin{aligned} d^\infty (\mathscr {F}(z(\nu ), \mathscr {F} y(\nu ))= & {} \sup _{\nu \in \mathbb {J}}\mathscr {H}^d\Big (\mathscr {F} (z(\nu ),\mathscr {F} (y(\nu )\Big )\\\le & {} \sup _{\nu \in \mathbb J} [a\mathbb {M}_b\mathbb {M}_s {\mathbb {L}}_f+a{\mathbb {L}}_c\mathbb {M}_b^2|{\mathscr {B}}\mathscr {E}\mathbb {M}_c\mathbb {M}_s {\mathbb {L}}_f| \mathscr {H}^d\Big (x(\tau ), y(\tau )\Big )\\\le & {} [b\mathbb {M}_b\mathbb {M}_s {\mathbb {L}}_f+b{\mathbb {L}}_c\mathbb {M}_b^2|\mathbb {B}\mathcal {E}\mathbb {M}_c\mathbb {M}_s {\mathbb {L}}_f|d^\infty (z(\nu ), y(\nu )). \end{aligned}$$

Hence,

$$\begin{aligned} \mathscr {H}^1\Big (\mathscr {F}(z(\nu ), \mathscr {F} y(\nu )\Big )= & {} \sup _{\nu \in \mathscr {J}}d^\infty (\mathscr {F} (z(\nu ),\mathscr {F} (y(\nu ))\\\le & {} \sup _{t\in \mathbb {J}} [a\mathbb {M}_b\mathbb {M}_s {\mathbb {L}}_f+a{\mathbb {L}}_c\mathbb {M}_b^2|\mathbb {B}\mathcal {E}|\mathbb {M}_c\mathbb {M}_s {\mathbb {L}}_fd^\infty (z(\nu ), y(\nu ))\\\le & {} \Delta \mathscr {H}^1\Big (z(\nu ), y(\nu )\Big ). \end{aligned}$$

Since $\Delta <1$, this show that the operator $\zeta$ is contraction on $\mathbb {E}_n$ and so, by Banach fixed point theorem, there exists a unique fixed point $z\in \mathscr {F}$ such that $\mathscr {F} ( z(\nu ))=z(\nu )$. This fixed point is the solution of (2.1)-(2.2). Thus Theorem 3.1 is proved.

### Case study on Sobolev type of FFDEs

Consider the fuzzy differential equation of the form with a non-local condition

3.1 $$\begin{aligned} (z(\nu ))^\prime\,= \; & {} \tilde{\varvec{3}}z(\nu )+\tilde{\varvec{3}}\nu z(\nu )^2 \end{aligned}$$

3.2 $$\begin{aligned} z(0)= & {} \sum _{i=1}^n c_i (\nu _i). \end{aligned}$$

The $\lambda$ level set of fuzzy number $\tilde{\varvec{3}}$: $[3]^\lambda =\,[\lambda +2,4-\lambda ]$. Now $\lambda$ level set of $\mathbb {F}(\nu , z(\nu ))=\,\tilde{\varvec{3}}\nu z(\nu )^2$ is

$$\begin{aligned}{}[\mathbb {F}(\nu , z(\nu )]^\lambda\,= \; & {} [\tilde{\varvec{3}}\nu z(\nu )^2]^\lambda \\= \; & {} \nu \Big [ (\lambda +2) z_l^\lambda (\nu +k)^2, (4-\lambda )z_r^\lambda (\nu +k)^2 \Big ] \end{aligned}$$

The $\lambda$- level set of $\displaystyle \sum _{i=1}^n c_i z(\nu _i)$:$\Big [\displaystyle \sum _{i=1}^n c_i z(\nu _i)\Big ]^\lambda =\Big [\sum _{i=1}^n c_i z_l^\lambda (\nu _i),\sum _{i=1}^n c_i x_r^\lambda (\nu _i)\Big ]$

$$\begin{aligned}{} & {} {\mathscr {H}^d\Big ([\mathbb {F}(\nu ,z(\nu ))]^\lambda ,[\mathbb {F}(\nu ,y(\nu ))]^\lambda \Big )}\\= & {} \mathscr {H}^d\Big (\nu [(\lambda +2)(z_l^\lambda (\nu ))^2, (4-\lambda )(z_r^\lambda (\nu ))^2],\\{} & {} t[(\lambda +2)(y_l^\lambda (\nu ))^2, (4-\lambda )(y_r^\lambda (\nu ))^2 ]\Big )\\= & {} t\max \{(\lambda +2)|(z_l^\lambda (\nu ))^2-(y_l^\lambda (\nu ))^2|,(4-\lambda )|(z_r^\lambda (\nu ))^2-(y_r^\lambda (\nu ))^2|\}\\= & {} \nu \max \{(\lambda +2)|z_l^\lambda (\nu )+y_l^\lambda (\nu )||z_l^\lambda (\nu )-y_l^\lambda (\nu )|,\\{} & {} (4-\lambda )|z_r^\lambda (\nu ))+(y_r^\lambda (\nu ))||z_r^\lambda (\nu )-y_r^\lambda (\nu )|\}\\\le & {} (4-\lambda )t|z_r^\lambda (\nu ))+y_r^\lambda (\nu )|\max \{|z_l^\lambda (\nu )-y_r^\alpha (\nu )|,|z_r^\alpha (\nu )-y_r^\alpha (\nu )|\}\\\le & {} (4-\lambda )b|z_r^\lambda (\nu ))+y_r^\lambda (\nu )|\max \{|z_l^\lambda (\nu )-y_l^\lambda (\nu )|,|z_r^\lambda (\nu )-y_r^\lambda (\nu )|\}\\\le & {} 4b|z_r^\lambda (\nu ))+y_r^\lambda (\nu )|\max \{|z_l^\lambda (\nu )-y_l^\lambda (\nu )|,|z_r^\lambda (\nu )-y_r^\lambda (\nu )|\}\\= & {} {\mathbb {L}}_f{\mathcal {H}}^d\Big ([z(\tau )]^\lambda ,[y(\tau )]^\lambda \Big ), \end{aligned}$$

where ${\mathbb {L}}_f=4b|x_r^\lambda (\nu ))+y_r^\lambda (\nu )|$ meets the inequality stated within the circumstance $(A_2)$.

$$\begin{aligned} \mathscr {H}^d\Big ([\sum _{i=1}^n c_i z(\nu _i)]^\lambda , [\sum _{i=1}^n c_i y(\nu _i)]^\lambda \Big ) \!\!\!= & {} \mathscr {H}^d\Big (\Big [\sum _{i=1}^n c_i z_l^\lambda (\nu _i),\sum _{i=1}^n c_i z_r^\lambda (\nu _i)\Big ], \Big [\sum _{i=1}^n c_i y_l^\lambda (\nu _i),\sum _{i=1}^n c_i y_r^\lambda (\nu _i)\Big ]\Big )\\\le & {} \mathbb {M}_c{\mathcal {H}}^d \Big ([z(\nu _i)]^\lambda ,y(\tau _i)]^\lambda \Big ), \end{aligned}$$

where $\mathbb {M}_c=|\displaystyle \sum _{i=1}^n c_i|$ satisfies the inequality which is given in condition $(A_2)$.

Thus, all conditions of Theorem 3.1 are satisfied. Hence the system (3.1)-(3.2) has a unique fuzzy solution.

## Fuzzy neutral integrodifferential equation

We shall explore at the non-linear neutral fuzzy integrodifferential equation of the form in this section.

4.1 $$\begin{aligned} \Big [\mathbb {B}z(\nu )+ \mathscr {P}(\nu , z(\nu ))\Big ]^\prime +\mathbb {A}z(\nu )= \; & {} \mathbb {F}(\nu ,z(\nu ))+\int _0^\nu {\mathscr {K}}(\nu ,\mu , z(\mu ))d\mu \nonumber \\{} & {} \; \hspace{3cm} \nu \in (0,a],\ \nu \ne \nu _k \end{aligned}$$

4.2 $$\begin{aligned} z(0)+\sum _{i=1}^n c_i z(\nu _i)= & {} z_0, \end{aligned}$$

where $\mathbb {A,B}:\mathbb {J}\rightarrow \mathbb {E}_n$ denotes a fuzzy coefficient and $\mathbb {J}=[0,a]$. The function $\mathbb {F}, \mathscr {P},\mathscr {K}: \mathbb {J}\times \mathbb {E}_n\rightarrow \mathbb {E}_n$ is nonlinear fuzzy function and $z_0$ is a initial value.

To prove the existence of (4.1)-(4.2), the following requirements must be fulfilled:

($$A_4$$)
The function $\mathscr {P}:\mathbb {J}\times \mathbb {E}_n\rightarrow \mathbb {E}_n$ satisfy the condition
  $$\begin{aligned} \mathscr {H}^d\Big ([\mathscr {P}(\nu , z(\nu ))]^\lambda ,[\mathscr {P}(\nu , y(\nu ))]^\lambda \Big )\le {\mathbb {L}}_p \mathscr {H}^d\Big (z(\nu ),y(\nu )\Big ). \end{aligned}$$
  There exists $${\mathbb {L}}_p\ge 0$$.
($$A_5$$)
The function ${\mathscr {K}}: \mathbb {J}\times \mathbb {E}_n\rightarrow \mathbb {E}_n$ satisfy the condition.
  $$\begin{aligned} \mathscr {H}^d\Big ([{\mathscr {K}}(\nu , z(\nu )]^\lambda ,[{\mathscr {K}}(\nu , y(\nu ))]^\lambda \Big )\le {\mathbb {L}}_k \mathscr {H}^d\Big (z(\nu ),y(\nu )\Big ). \end{aligned}$$
  There exists $${\mathbb {L}}_k\ge 0.$$
($$A_6$$)
For convenience
  $$\begin{aligned} \mathbb {M}_b |\mathbb {B}\mathcal {E}z_0|\mathbb {M}_s+ & {} \mathbb {M}_c \mathbb {M}_b^2|\mathbb {B}\mathcal {E}|{\mathbb {L}}_p + \mathscr {M}_s[\mathscr {L}_p+a\mathscr {M}_s(\mathscr {L}_p+\mathscr {L}_f+a\mathscr {L}_k]\\+ & {} \mathscr {M}_b \mathscr {M}_s[\mathscr {L}_p+\mathscr {L}_f+a\mathscr {L}_k+b(\mathscr {L}_p]\le \mathcal {r}. \end{aligned}$$

In this section, we’ll suppose that there is an operator $\mathbb {E}$ on $\mathbb {E}_n$, which can be found using the formula

$$\begin{aligned} \mathcal {E}=\Big [\mathbb I+\sum _{i=1}^n c_i \mathbb {B}^{-1}{\mathscr {S}}(\nu _i)\mathbb {B} \Big ]^{-1} \end{aligned}$$

with

$$\begin{aligned} \mathcal {E}\Big \{\mathbb {B}^{-1} \mathscr {P}(\nu ,z(\nu ))- & {} \mathbb {B}^{-1} {\mathscr {S}}(\nu _i)\mathscr {P}(0,z(0))+ \int _0^{\nu _i} \mathbb {A}{\mathscr {S}}(\nu _i-\mu )\mathbb {B}^{-1}\mathscr {P}(\mu , z(\mu ))d\mu \Big \}\\+ & {} \int _0^{\nu _i} \mathbb {B}^{-1} {\mathscr {S}}(\nu _i-\mu )\Big [ \mathbb {F}(\mu , z(\mu ))+\int _0^\mu {\mathscr {K}}(\mu , \nu , z(\nu ))d\nu \Big ]d\mu \Big \} \in \mathbb {E}_n. \end{aligned}$$

### Definition 4.1

An expression for the integral equation $z(\nu )$

4.3 $$\begin{aligned} z(\nu )\!\!\!\,= & {} \mathbb {B}^{-1} {\mathscr {S}}(\nu )\mathbb {B}\mathbb {E}z_0 +\sum _{i=1}^n c_i \mathbb {B}^{-1}{\mathscr {S}}(\nu ) \mathbb {B}\mathcal {E}\Big \{ \mathbb {B}^{-1} \mathscr {P}(\nu , z(\nu )-\mathbb {B}^{-1}{\mathscr {S}}(\nu _i)\mathscr {P}(0,z(0)\Big \}\nonumber \\{} & {} +\sum _{i=1}^n c_i \mathbb {B}^{-1}{\mathscr {S}}(\nu ) \mathbb {B}\mathcal {E}\Big \{ \int _0^{\nu _i}\mathbb {B}^{-1} {\mathscr {S}}(\nu -\mu )\Big [ \mathbb {A}\mathscr {P}(\mu , z(\mu )+\mathbb {F}(\mu ,z(\mu )\nonumber )\\{} & {} +\int _0^\mu {\mathscr {K}}(\mu , \nu , z(\nu ))d\nu \Big ] d\mu \Big \} +\mathbb {B}^{-1} {\mathscr {S}}(\nu )\mathscr {P}(0,z(0))-\mathbb {B}^{-1} \mathscr {P}(\nu , z(\nu ))\nonumber \\{} & {} \!\!+ \!\!\int _0^\nu \!\! {\mathscr {S}}(\nu -\mu ) \mathbb {B}^{-1} \Big [ \mathbb {A}\mathscr {P}(\mu , z(\mu ))+ \mathbb {F}(\mu , z(\mu ))+\int _0^\mu \!\!{\mathscr {K}}(\mu , \nu , z(\nu ))d\nu \Big ] d\mu . \end{aligned}$$

is said to be a fuzzy solution of (4.1, 4.2) on $\mathbb {E}_n$.

### Remark 4.1

The fuzzy functional neutral integrodifferential equation (4.1, 4.2) has a fuzzy solution that satisfies (4.3).

$$\begin{aligned} z(0)\,= \; & {} \mathbb {E}z_0 +\sum _{i=1}^n c_i \mathcal {E} \Big \{ \mathbb {B}^{-1} \mathscr {P}(\nu , z(\nu ))-\mathbb {B}^{-1} {\mathscr {S}}(\nu _i) \mathcal {E}(0,z(0))\}\\{} & {} \times \Big [\mathbb {A}\mathscr {P}(\mu , z_\mu (\nu ))+\mathbb {F}(\mu , z(\mu ))+\int _0^\mu {\mathscr {K}}(\mu , \nu , z(\nu )) d\nu \Big ]d\mu \Big \} \ \ \ \ \ \text{ and } \\ z(\nu _j)\,= \; & {} \mathbb {B}^{-1}{\mathscr {S}}(\nu _j) \mathcal {E}z_0 +\sum _{i=1}^n c_i\mathbb {B}^{-1}{\mathscr {S}}(\nu _j) \mathcal {E} \Big \{ \mathbb {B}^{-1} \mathscr {P}(\nu , z(\nu ))-\mathbb {B}^{-1} {\mathscr {S}}(\nu _i) \mathcal {E}(0,z(0))\}\\+ & {} \sum _{i=1}^n c_i \mathbb {B}^{-1}{\mathscr {S}}(\nu _j) \mathcal {E} \Big \{\int _0^{\nu _i}{\mathscr {S}}(\nu _i-\mu )\mathbb {B}^{-1}\Big [\mathbb {A}\mathscr {P}(\mu , z_\mu (\nu ))+\mathbb {F}(\mu , z_\mu (\mu ))\\+ & {} \int _0^\mu {\mathscr {K}}(\mu , \nu , z(\nu )) d\nu \Big ]d\mu \Big \}+\mathbb {B}^{-1}{\mathscr {S}}(\nu _j) \mathscr {P}(0,z(0))\\- & {} \mathbb {B}^{-1}\mathscr {P}(\nu _j,z_\nu (\nu _j))\int _0^{\nu _j} {\mathscr {S}}(\nu _j-\mu ) \mathbb {B}^{-1} \Big [\mathbb {A}\mathscr {P}(\mu , z_\mu (\mu ))\\+ & {} \mathbb {F}(\mu ,z_\mu (\mu ))+\int _0^\mu {\mathscr {K}}(\mu , \nu , z(\nu ))d\nu \Big ]d\mu . \end{aligned}$$

Therefore,

$$\begin{aligned}{} & {} {z(0)+\sum _{j=1}^n c_j z(\nu _j)}\\\,= \; & {} \mathbb {B}^{-1}{\mathscr {S}}(\nu _j) \mathcal {E}z_0 +\sum _{i=1}^n c_i\mathbb {B}^{-1}{\mathscr {S}}(\nu _j) \mathcal {E} \Big \{ \mathbb {B}^{-1} \mathscr {P}(\nu , z(\nu ))-\mathbb {B}^{-1} {\mathscr {S}}(\nu _i) \mathcal {E}(0,z(0))\}\\+ & {} \sum _{i=1}^n c_i \mathbb {B}^{-1}{\mathscr {S}}(\nu _j) \mathcal {E} \Big \{\int _0^{\nu _i}{\mathscr {S}}(\nu _i-\mu )\mathbb {B}^{-1}\Big [\mathbb {A}\mathscr {P}(\mu , z_\mu (\nu ))+\mathbb {F}(\mu , z_\mu (\mu ))\\+ & {} \int _0^\mu {\mathscr {K}}(\mu , \nu , z(\nu )) d\nu \Big ]d\mu \Big \} +\mathbb {B}^{-1}{\mathscr {S}}(\nu _j) \mathscr {P}(0,z(0))-\mathbb {B}^{-1}\mathscr {P}(\nu _j,z_\nu (\nu _j))\\\times & {} \int _0^{\nu _j} {\mathscr {S}}(\nu _j-\mu ) \mathbb {B}^{-1} \Big [\mathbb {A}\mathscr {P}(\mu , z_\mu (\mu ))+\mathbb {F}(\mu ,z_\mu (\mu ))+\int _0^\mu {\mathscr {K}}(\mu , \nu , z(\nu ))d\nu \Big ]d\mu . \end{aligned}$$

### Theorem 4.2

If the assumptions $(A_1)$-$(A_6)$ are true, then (4.1)-(4.2) offers an ambiguous conclusion on $\mathbb {J}$.

### Proof

Let $\mathcal F_1$ be the subset of $\mathbb {C}(\mathbb J, \mathbb {E}_n)$ defined by

$$\begin{aligned} \mathcal F_1\,=\,\{z: z(\nu )\in \mathbb {E}_n, |z(\nu )|\le r, \ \text {for}\ \ t\in \mathbb J \}. \end{aligned}$$

We define a mapping $\tilde{\mathscr {F}}:\mathcal F_1 \rightarrow \mathcal F_1$ by

$$\begin{aligned} (\tilde{\mathscr {F}} z)(\nu )&=\mathbb {B}^{-1}{\mathscr {S}}(\nu )[\mathbb {B}^{-1}{\mathscr {S}}(\nu )\mathbb {B}\mathcal {E}z_0-\sum ^n_{i=1}c_i\mathbb {B}^{-1}{\mathscr {S}}(\nu )\mathbb {B}\mathcal {E} \\&\quad \times \Big \{\int ^{\nu _i}_0 \mathbb {B}^{-1}{\mathscr {S}}(\nu _i-\mu )\mathbb {F}(\mu ,z(\mu ))d\mu +\int ^t_0 {\mathscr {S}}(\nu -\mu )\mathbb {B}^{-1}\mathbb {F}(\mu ,z(\mu ))d\mu . \end{aligned}$$

First to show that the operator $\tilde{\mathscr {F}}$ maps $\mathcal F_1$ into itself. Now

$$\begin{aligned} |(\tilde{\mathscr {F}} z)(\nu )|= \; & {} |\mathbb {B}^{-1} {\mathscr {S}}(\nu )\mathbb {B}\mathcal {E}z_0 +\sum _{i=1}^n c_i \mathbb {B}^{-1}{\mathscr {S}}(\nu ) \mathbb {B}\mathcal {E} \Big \{ \mathbb {B}^{-1} \mathscr {P}(\nu , z(\nu )-\mathbb {B}^{-1}{\mathscr {S}}(\nu _i)\mathscr {P}(0,z(0)\Big \}\nonumber \\{} & {} +\sum _{i=1}^n c_i \mathbb {B}^{-1}{\mathscr {S}}(\nu ) \mathbb {B}\mathcal {E}\Big \{ \int _0^{\nu _i}\mathbb {B}^{-1} {\mathscr {S}}(\nu -\mu )\Big [ \mathbb {A}\mathscr {P}(\mu , z(\mu )+\mathbb {F}(\mu ,z(\mu ))\\{} & {} + \int _0^\mu {\mathscr {K}}(\mu , \nu , z(\nu ))d\nu \Big ] d\mu \Big \} +\mathbb {B}^{-1} {\mathscr {S}}(\nu )\mathscr {P}(0,z(0))-\mathbb {B}^{-1} \mathscr {P}(\nu , z(\nu ))\nonumber \\{} & {} + \int _0^\nu {\mathscr {S}}(\nu -\mu ) \mathbb {B}^{-1} \Big [ \mathbb {A}\mathscr {P}(\mu , z(\mu ))+ \mathbb {F}(\mu , z(\mu ))+\int _0^\mu {\mathscr {K}}(\mu , \nu , z(\nu ))d\nu \Big ] d\mu |\\\le & {} |\mathbb {B}^{-1} {\mathscr {S}}(\nu )\mathbb {B}\mathcal {E}z_0| +|\sum _{i=1}^n c_i \mathbb {B}^{-1}{\mathscr {S}}(\nu ) \mathbb {B}\mathcal {E} \Big \{ \mathcal {B}^{-1} \mathscr {P}(\nu , z(\nu )-\mathbb {B}^{-1}{\mathscr {S}}(\nu _i)\mathscr {P}(0,z(0)\Big \}|\\{} & {} +|\quad \sum _{i=1}^n c_i \mathbb {B}^{-1}{\mathscr {S}}(\nu ) \mathbb {B}\mathcal {E}\Big \{ \int _0^{\nu _i}\mathbb {B}^{-1} {\mathscr {S}}(\nu -\mu )\Big [ \mathbb {A}\mathscr {P}(\mu , z(\mu )+\mathbb {F}(\mu ,z(\mu )\\{} & {} +\quad \int _0^\mu {\mathscr {K}}(\mu , \nu , z(\nu ))d\nu \Big ] d\mu \Big \}| +|\mathbb {B}^{-1} {\mathscr {S}}(\nu )\mathscr {P}(0,z(0))-\mathbb {B}^{-1} \mathscr {P}(\nu , z(\nu ))\nonumber \\{} & {} + \int _0^\nu {\mathscr {S}}(\nu -\mu ) \mathbb {B}^{-1} \Big [ \mathbb {A}\mathscr {P}(\mu , z(\mu ))+ \mathbb {F}(\mu , z(\mu ))+\int _0^\mu {\mathscr {K}}(\mu , \nu , z(\nu ))d\nu \Big ] d\mu |\\\le & {} \mathbb {M}_b |{\mathscr {B}}\mathscr {E}x_0|\mathbb {M}_s+ \mathbb {M}_c \mathbb {M}_b^2|{\mathscr {B}}\mathscr {E}|{\mathbb {L}}_p\mathscr {M}_s[\mathscr {L}_p+b\mathscr {M}_s[\mathscr {L}_p+\mathscr {L}_f+a\mathscr {L}_k]\\{} & {} + \mathscr {M}_b \mathscr {M}_s[\mathscr {L}_p+\mathscr {L}_f+b\mathscr {L}_k+a\mathscr {L}_p]. \end{aligned}$$

From the assumption $(A_6),\ |(\tilde{\mathscr {F}} z)(\nu )|\le \eta$. Therefore $\tilde{\mathscr {F}}$ maps $\mathcal {F}_1$ into itself. Moreover, if $z, y \in \mathcal {F}_1,$ then

$$\begin{aligned}{} & {} { \mathscr {H}^d\Big ([\tilde{\mathscr {F}} z)(\nu )]^\lambda , [(\tilde{\mathscr {F}}z)(\nu )]^\lambda \Big )}\\= & {} \mathscr {H}^d\Big ( \Big [ {\mathscr {B}}^{-1} {\mathscr {S}}(\nu )\mathbb {B}\mathcal {E}z_0 +\sum _{i=1}^n c_i \mathbb {B}^{-1}{\mathscr {S}}(\nu ) {\mathscr {B}}\mathscr {E}\Big \{ {\mathscr {B}}^{-1} \mathscr {P}(\tau , x(\tau )-{\mathscr {B}}^{-1}{\mathscr {S}}(\tau _i)\mathscr {P}(0,x(0)\Big \}\\{} & {} + \ \sum _{i=1}^n c_i \mathbb {B}^{-1}{\mathscr {S}}(\nu ) \mathbb {B}\mathcal {E}\Big \{ \int _0^{\nu _i}\mathbb {B}^{-1} {\mathscr {S}}(\nu -\mu )\Big [ \mathbb {A}\mathscr {P}(\mu , z(\mu )+\mathbb {F}(\mu ,z(\mu ))\\{} & {} + \int _0^\mu {\mathscr {K}}(\mu , \nu , z(\nu ))d\nu \Big ] d\mu \Big \} +\mathbb {B}^{-1} {\mathscr {S}}(\nu )\mathscr {P}(0,z(0))-\mathbb {B}^{-1} \mathbb {P}(\nu , z(\nu )) + \int _0^\nu {\mathscr {S}}(\nu -\mu ) \\{} & {} \times \mathbb {B}^{-1} \Big [ \mathbb {A}\mathscr {P}(\mu , z(\mu ))+ \mathbb {F}(\mu , z(\mu ))+\int _0^\mu {\mathscr {K}}(\mu , \nu , z(\nu ))d\nu \Big ] d\mu \Big ]^\lambda ,\Big [ \mathbb {B}^{-1} {\mathscr {S}}(\nu )\mathbb {B}\mathcal {E}y_0 \\{} & {} +\sum _{i=1}^n c_i \mathbb {B}^{-1}{\mathscr {S}}(\nu ) \mathbb {B}\mathcal {E}\Big \{ \mathbb {B}^{-1} \mathscr {P}(\nu , y(\nu )-\mathbb {B}^{-1}{\mathscr {S}}(\nu _i)\mathscr {P}(0,y(0)\Big \}\\{} & {} + \sum _{i=1}^n c_i \mathbb {B}^{-1}{\mathscr {S}}(\nu ) \mathbb {B}\mathcal {E}\Big \{ \int _0^{\nu _i}\mathbb {B}^{-1} {\mathscr {S}}(\nu -\mu )\Big [ \mathbb {A}\mathscr {P}(\mu , y(\mu )+\mathbb {F}(\mu ,y(\mu ))\\{} & {} + \int _0^\mu {\mathscr {K}}(\mu , \nu , y(\nu ))d\nu \Big ] d\mu \Big \} +\mathbb {B}^{-1} {\mathscr {S}}(\nu )\mathscr {P}(0,y(0))-\mathbb {B}^{-1} \mathscr {P}(\nu , y(\nu )) + \int _0^\nu {\mathscr {S}}(\nu -\mu ) \\{} & {} \times \mathbb {B}^{-1} \Big [ \mathbb {A}\mathscr {P}(\mu , y(\mu ))+ \mathbb {F}(\mu , y(\mu ))+\int _0^\mu {\mathscr {K}}(\mu , \nu , y(\nu ))d\tau \Big ] d\mu \Big ]^\lambda \Big )\\\le & {} \mathbb {M}_b |\mathbb {B}\mathcal {E}x_0|\mathbb {M}_s+ \mathbb {M}_c \mathbb {M}_b^2|{\mathscr {B}}\mathcal {E}|{\mathbb {L}}_p\mathscr {M}_s[\mathscr {L}_p+b\mathscr {M}_s[\mathscr {L}_p+\mathscr {L}_f+a\mathscr {L}_k]\\{} & {} +\quad \mathscr {M}_b \mathscr {M}_s[\mathscr {L}_p+\mathscr {L}_f+a\mathscr {L}_k+a\mathscr {L}_p] \le \mathcal {r} \mathscr {H}^d\Big ([z(\nu )]^\lambda ,[y(\nu )]^\lambda \Big ), \end{aligned}$$

where $$\mathcal {x}= \mathbb {M}_b |{\mathscr {B}}\mathscr {E}z_0|\mathbb {M}_s+ \mathbb {M}_c \mathbb {M}_b^2|{\mathscr {B}}\mathscr {E}|{\mathbb {L}}_p\mathscr {M}_s[\mathscr {L}_p+b\mathscr {M}_s[\mathscr {L}_p+\mathscr {L}_f+b\mathscr {L}_k] \mathscr {M}_b \mathscr {M}_s[\mathscr {L}_p+\mathscr {L}_f+b\mathscr {L}_k+b\mathscr {L}_p].$$

Now,

$$\begin{aligned} d^\infty \Big ([\tilde{\mathscr {F}} z(\nu )]^\lambda ,[\tilde{\mathscr {F}} y(\nu )]^\lambda \Big )&\le \quad \sup _{\lambda \in (0,1)}{\mathcal {H}}^d ([\mathscr {F} z(\nu )]^\lambda ,[\mathscr {F} y(\nu )]^\lambda )\\&\le \quad \mathcal {x}d^\infty \Big (z(\nu ),y(\nu )\Big ). \end{aligned}$$

Therefore,

$$\begin{aligned} \mathscr {H}^1\Big ([\tilde{\mathscr {F}} z(\nu )]^\lambda ,[\tilde{\mathscr {F}}y(\nu )]^\lambda \Big )&\le \quad \sup _{\lambda \in (0,1)}d^\infty ([\mathscr {F}z(\nu )]^\lambda ,[\mathscr {F} y(\nu )]^\lambda )\\&\le \quad \mathcal {x} {\mathcal {H}}^1 \Big (z(\nu ),y(\nu )\Big ). \end{aligned}$$

Since $\mathcal {x}<1,$ the above equation, demonstrate the contraction associated with the operator $\tilde{\mathscr {F}}$ over $\mathbb {E}_n$ and hence by Banach fixed point theorem there exists a unique fixed point $z \in \mathcal {F}_1$ such that $(\tilde{\mathscr {F}} z)(\nu )=z(\nu ).$ This fixed point is then the solution of the problem (4.1, 4.2).

**Case study**: Consider the non-local condition on the fuzzy neutral indegrodifferential equation

4.4 $$\begin{aligned} (z(\nu )-\varvec{4}z(\nu ))^\prime= & {} \tilde{\varvec{3}}x(\tau )+\tilde{\varvec{3}}\tau x^2(\tau )+\tilde{\varvec{3}}\tau x^2(\tau ); \end{aligned}$$

4.5 $$\begin{aligned} x(0)= & {} \sum _{i=1}^n c_i (\tau _i). \end{aligned}$$

The $\lambda$ level set of fuzzy number $\tilde{\varvec{3}}$: $[3]^\lambda =[\lambda +2,4-\lambda ]$. Now $\lambda$ level set of $\mathscr {F}(\tau , x(\tau ))=\tilde{\varvec{3}}\tau x(\tau )^2$ is

$$\begin{aligned}{}[\mathscr {F}(\nu , z(\nu ))]^\lambda= & {} [\tilde{\varvec{3}}\nu z(\nu )^2]^\lambda \\= & {} \nu \Big [ (\lambda +2) z_l^\lambda (\nu )^2, (4-\lambda )z_r^\lambda (\nu )^2 \Big ]. \end{aligned}$$

The $\lambda$- level set of $\sum _{i=1}^n c_i z(\nu _i)$:$\Big [\sum _{i=1}^n c_i z(\nu _i)\Big ]^\lambda =\Big [\sum _{i=1}^n c_i z_l^\lambda (\nu _i),\sum _{i=1}^n c_i x_r^\lambda (\nu _i)\Big ]$

$$\begin{aligned}{} & {} {\mathscr {H}^d\Big ([\mathbb {F}(\nu ,z(\nu ))]^\lambda ,[\mathbb {F}(\nu ,y(\nu ))]^\lambda \Big )}\\=\, & {} \mathscr {H}^d\Big (\nu [(\lambda +2)(z_l^\lambda (\nu ))^2, (4-\lambda )(z_r^\lambda (\nu ))^2],\\{} & {} t[(\lambda +2)(y_l^\lambda (\nu ))^2, (4-\lambda )(y_r^\lambda (\nu ))^2 ]\Big )\\=\, & {} t\max \{(\lambda +2)|(z_l^\lambda (\nu ))^2-(y_l^\lambda (\nu ))^2|,(4-\lambda )|(z_r^\lambda (\nu ))^2-(y_r^\lambda (\nu ))^2|\}\\=\, & {} \nu \max \{(\lambda +2)|z_l^\lambda (\nu )+y_l^\lambda (\nu )||z_l^\lambda (\nu )-y_l^\lambda (\nu )|,\\{} & {} (4-\lambda )|z_r^\lambda (\nu ))+(y_r^\lambda (\nu ))||z_r^\lambda (\nu )-y_r^\lambda (\nu )|\}\\\le & {} (4-\lambda )t|z_r^\lambda (\nu ))+y_r^\lambda (\nu )|\max \{|z_l^\lambda (\nu )-y_r^\lambda (\nu )|,|z_r^\lambda (\nu )-y_r^\lambda (\nu )|\}\\\le & {} (4-\lambda )b|z_r^\lambda (\nu ))+y_r^\lambda (\nu )|\max \{|z_l^\lambda (\nu )-y_l^\lambda (\nu )|,|z_r^\lambda (\nu )-y_r^\lambda (\nu )|\}\\\le & {} 4b|z_r^\lambda (\nu ))+y_r^\lambda (\nu )|\max \{|z_l^\lambda (\nu )-y_l^\lambda (\nu )|,|z_r^\lambda (\nu )-y_r^\lambda (\nu )|\}\\=\, & {} {\mathbb {L}}_f \mathscr {H}^d\Big ([z(\nu )]^\lambda ,[y(\nu )]^\lambda \Big ), \end{aligned}$$

where ${\mathbb {L}}_f\,=\,4b|z_r^\lambda (\nu ))+y_r^\lambda (\nu )|$ satisfies the inequality which is given in condition $(A_2)$.

$$\begin{aligned} \mathscr {H}^d\Big ([\sum _{i=1}^n c_i z(\nu _i)]^\lambda , [\sum _{i=1}^n c_i y(\nu _i)]^\lambda \Big ) =\, & {} {\mathcal {H}}^d\Big (\Big [\sum _{i=1}^n c_i x_l^\lambda (\nu _i),\sum _{i=1}^n c_i x_r^\lambda (\nu _i)\Big ],\Big [\sum _{i=1}^n c_i y_l^\lambda (\nu _i),\sum _{i=1}^n c_i y_r^\lambda (\nu _i)\Big ]\Big )\\\le & {} \mathbb {M}_c\mathscr {H}^d \Big ([z(\nu _i)]^\lambda ,y(\nu _i)]^\lambda \Big ), \end{aligned}$$

where $\mathbb {M}_c\,=\,|\sum _{i=1}^n c_i|$ satisfies the inequality which is given in condition $(A_2)$.

$$\begin{aligned} \mathscr {H}^d\Big ([\mathscr {P}(\nu , z(\nu ))]^\lambda ,[\mathscr {P}(\nu ,y(\nu ))]^\lambda \Big )\,=\, & {} \mathscr {H}^d\Big (\nu [(\lambda +3)(z_l^\lambda (\nu )^2), (5-\lambda )(z_r^\lambda (\nu )^2)],\\{} & {} \tau [(\lambda +1)(y_l^\lambda (\nu )^2), (3-\lambda )(y_r^\lambda (\nu ))^2]\Big )\\= & {} \nu \max \{(\lambda +1)|(z_l^\lambda (\nu ))-(y_l^\lambda (\nu ))|,(3-\lambda )|(z_r^\lambda (\nu ))-(y_r^\lambda (\nu ))|\}\\\le & {} 5b \mathscr {H}^d \Big ([z(\nu _i]^\lambda ,y(\nu _i]^\lambda \Big )={\mathbb {L}}_p \mathscr {H}^d \Big ([z(\nu _i]^\lambda ,y(\nu _i)]^\lambda \Big ), \end{aligned}$$

where, ${\mathbb {L}}_p=5b|x_r^\lambda (\tau ))+y_r^\lambda (\tau )|$ satisfies the inequality which is given in condition $(A_4)$.

$$\begin{aligned} \mathscr {H}^d\Big ([\int _0^\nu {\mathscr {K}}(\nu ,\mu , z(\mu ))]^\lambda ,[\int _0^\nu {\mathscr {K}}(\nu ,\mu , z(\mu ))]^\lambda \Big )&= \mathscr {H}^d\Big (\nu [(\lambda +2)(z_l^\lambda (\nu )^2) , (4-\lambda )(z_r^\lambda (\nu )^2)],\\&\quad \tau [(\lambda +1)(y_l^\lambda (\nu )^2) , (3-\lambda )(y_r^\lambda (\nu ))^2]\Big )\\&=\quad \tau \max \{(\lambda +2)|(z_l^\lambda (\nu ))-(y_l^\lambda (\nu ))|,\\&\quad (4-\lambda )|(z_r^\lambda (\nu ))-(y_r^\lambda (\nu ))|\}\\&\le \quad 4b \mathscr {H}^d ([z(\nu _i]^\lambda ,y(\nu _i]^\lambda )={\mathbb {L}}_k \mathscr {H}^d \Big ([z(\nu _i)]^\lambda ,y(\nu _i)]^\lambda \Big ), \end{aligned}$$

where, ${\mathbb {L}}_k=4b|z_r^\lambda (\nu ))+y_r^\lambda (\nu )|$ satisfies the inequality which is given in condition $(A_5)$.

Thus, all conditions of Theorem 4.1 are satisfied. Hence the system (4.4, 4.5) has a unique fuzzy solution.

## Fuzzy impulsive neutral integrodifferential equation

Take into account the following: Neutral integrodifferential equation with fuzziness

5.1 $$\begin{aligned}{}[\mathbb {B}z(\nu )+ \mathscr {P}(\nu , z(\nu ))]^\prime +\mathbb {A}z(\nu )= & {} \mathbb {F}(\nu ,z(\nu ))+\int _0^\nu {\mathscr {K}}(\nu ,\mu , z(\mu ))d\mu \nonumber \\{} & {} \; \hspace{3cm} \nu \in (0,z], \nu \ne \nu _k \end{aligned}$$

5.2 $$\begin{aligned} z(0)+\sum _{i=1}^n c_i z(\nu _i)= \; & {} z_0 \end{aligned}$$

5.3 $$\begin{aligned} \Delta z(\nu _k)= & {} {\mathcal {I}}_k(z_{\nu _k}); k=1,2,...,m, \end{aligned}$$

where $\mathbb {A,B}:\mathbb {J}\rightarrow \mathbb {E}_n$ denotes a fuzzy coefficient and $\mathbb {J}=[0,a]$, $\mathbb {E}_n$ is the collection of all upper semi continuous convex normal fuzzy numbers with bounded $\lambda -$level intervals. The function $\mathbb {F}, \mathscr {P},\mathscr {K}:\mathbb J\times \mathbb {E}_n\rightarrow \mathbb {E}_n$ is nonlinear fuzzy function and $z_0$ is a initial value and ${\mathcal {I}}_kz(\nu _k)=\Delta z(\nu _k)=z(\nu _k^+)-z(\nu _k^-)\in \mathbb {E}_n$. Denote $\mathbb J_0=[0, \nu _1],\;\nu _k=(\nu _k, \nu _{k+1}],\;k=1, 2, \ldots , m$ and define the following space:

Let $\mathscr{P}\mathscr{C}([0, a], \mathbb X)=\{ z: z$ is a function from [0, *a*] into $\mathbb X$ such that $z(\nu )$ is continuous at $\nu \ne \nu _k$ and left continuous at $\nu =\nu _i$ and the right limit $z(\nu _k^+)$ exists for $k=1,2,\ldots ,m$}. Similarly as in ([33]), we see that $\mathscr{P}\mathscr{C}([0,a], \mathbb X)$ is a Banach space with norm

$$\begin{aligned} \Vert z\Vert _{\mathscr{P}\mathscr{C}}=\sup _{\nu \in [0,a]}\Vert z(\nu )\Vert . \end{aligned}$$

To demonstrate the system’s existence (5.1, 5.2, 5.3). The following requirements must be fulfilled in order for it to work:

($$A_7$$)
  $$\begin{aligned} {\mathcal {H}}^d\Big ([{\mathcal {I}}_k(z(\nu _k^-))]^\lambda , [{\mathcal {I}}_k(y(\nu _k^-))]^\lambda \Big )\le & {} l_i \mathscr {H}^d\Big ([z(\nu ))]^\lambda , [y(\nu ))]^\lambda \Big ),\\ \sum _{i=1}^{k}l_i= & {} \mathcal {L}_i. \end{aligned}$$

In this part, we presume that an operator $\mathcal E$ exists on $\mathbb E_n$, which is described by the formula.

$$\begin{aligned} \mathcal {E}=\Big [\mathbb I+\sum _{i=1}^n c_i \mathbb {B}^{-1}{\mathscr {S}}(\nu _i)\mathbb {B} \Big ]^{-1} \end{aligned}$$

with

$$\begin{aligned} \mathcal {E}\Big \{\mathbb {B}^{-1} \mathscr {P}(\nu ,z(\nu ))- & {} \mathbb {B}^{-1} {\mathscr {S}}(\nu _i)\mathscr {P}(0,z(0))+ \int _0^{\nu _i} \mathbb {A}{\mathscr {S}}(\nu _i-\mu )\mathbb {B}^{-1}\mathscr {P}(\mu , z(\mu ))d\mu \Big \}\\+ & {} \int _0^{\nu _i} \mathbb {B}^{-1} {\mathscr {S}}(\nu _i-\mu )\Big [ \mathbb {F}(\mu , z(\mu ))+\int _0^\mu {\mathscr {K}}(\mu , \nu , z(\nu ))d\nu \Big ]d\mu \Big \} \in \mathbb {E}_n. \end{aligned}$$

### Definition 5.1

The integral equation’s *z* fuzzy solution

5.4 $$\begin{aligned} z(\nu )= \,\Psi& {} \mathbb {B}^{-1} {\mathscr {S}}(\nu )\mathbb {B}\mathcal {E}z_0 +\sum _{i=1}^n c_i \mathbb {B}^{-1}{\mathscr {S}}(\nu ) \mathbb {B}\mathcal {E}\nonumber \\\times & {} \Big \{ \mathbb {B}^{-1} \mathscr {P}(\nu , z(\nu )-\mathbb {B}^{-1}{\mathscr {S}}(\nu _i)\mathscr {P}(0,z(0))-\sum _{0<\nu _k<\nu }\mathbb {B}^{-1}{\mathscr {S}}(\nu _k-\nu _i){\mathcal {I}}_k(z(\nu _k))\Big \}\nonumber \\+ & {} \sum _{i=1}^n c_i \mathbb {B}^{-1}{\mathscr {S}}(\nu ) \mathbb {B}\mathcal {E}\Big \{ \int _0^{\nu _i}\mathbb {B}^{-1} {\mathscr {S}}(\nu -\mu )\Big [ \mathbb {A}\mathscr {P}(\mu , z(\mu )+\mathbb {F}(\mu ,z(\mu ))\nonumber )\\+ & {} \int _0^\mu {\mathscr {K}}(\mu , \nu , z(\nu ))d\nu \Big ] d\mu \Big \} +\mathbb {B}^{-1} {\mathscr {S}}(\nu )\mathscr {P}(0,z(0))-\mathbb {B}^{-1} \mathscr {P}(\nu , z(\nu ))\nonumber \\+ & {} \int _0^\nu {\mathscr {S}}(\nu -\mu )\mathbb {B}^{-1} \Big [ \mathbb {A}\mathscr {P}(\mu , z(\mu ))+ \mathbb {F}(\mu , z(\mu ))+\int _0^\mu {\mathscr {K}}(\mu , \nu , z(\nu ))d\nu \Big ] d\mu \nonumber \\- & {} \sum _{0<\nu _k<\nu }\mathbb {B}^{-1}{\mathscr {S}}(\nu _k-\nu _i){\mathcal {I}}_k(z(\nu _k)) \end{aligned}$$

is said to be a solution of (5.1, 5.2, 5.3) on $\mathbb {J}$.

### Remark 5.1

The fuzzy neutral integrodifferential equation (5.1, 5.2, 5.3) of the Sobolev type satisfies (5.3).

$$\begin{aligned} z(0)= \; & {} \mathcal {E}z_0 +\sum _{i=1}^n c_i \mathcal {E} \Big \{ \mathbb {B}^{-1} \mathscr {P}(\nu , z(\nu ))-\mathbb {B}^{-1} {\mathscr {S}}(\nu _i) \mathscr {P}(0,z(0))-\sum _{0<\nu _k<\nu }\mathbb {B}^{-1}{\mathscr {S}}(\nu _k-\nu _i){\mathcal {I}}_k(z(\nu _k))\}\\+ & {} \sum _{i=1}^n c_i \mathcal {E} \Big \{\int _0^{\nu _i}{\mathscr {S}}(\nu _i-\mu )\mathbb {B}^{-1}\Big [\mathbb {A}\mathscr {P}(\mu , z_\mu (\nu ))+f(\mu , z(\mu ))+\int _0^\mu {\mathscr {K}}(\mu , \nu , z(\nu )) d\nu \Big ]d\mu \Big \} \end{aligned}$$

and

$$\begin{aligned} z(\nu _j)= \; & {} \mathbb {B}^{-1}{\mathscr {S}}(\nu _j) \mathcal {E}z_0 +\sum _{i=1}^n c_i\mathbb {B}^{-1}{\mathscr {S}}(\nu _j) \mathcal {E} \Big \{ \mathbb {B}^{-1} \mathscr {P}(\nu , z(\nu ))-\mathbb {B}^{-1} {\mathscr {S}}(\nu _i) \mathcal {E}(0,z(0))\}\\- & {} \sum _{0<\nu _k<\nu }\mathbb {B}^{-1}{\mathscr {S}}(\nu _j-\nu _i){\mathcal {I}}_k(z(\nu _j))\\+ & {} \sum _{i=1}^n c_i \mathbb {B}^{-1}{\mathscr {S}}(\nu _j) \mathcal {E} \Big \{\int _0^{\nu _i}{\mathscr {S}}(\nu _i-\mu )\mathbb {B}^{-1}\Big [\mathbb {A}\mathscr {P}(\mu , z_\mu (\nu ))+\mathbb {F}(\mu , z_\mu (\mu ))\\+ & {} \int _0^\mu {\mathscr {K}}(\mu , \nu , z(\nu )) d\nu \Big ]d\mu \Big \}+\mathbb {B}^{-1}{\mathscr {S}}(\nu _j) \mathscr {P}(0,z(0))-\mathbb {B}^{-1}\mathscr {P}(\nu _j,z_\nu (\nu _j))\int _0^{\nu _j} {\mathscr {S}}(\nu _j-\mu )\\\times & {} \mathbb {B}^{-1} \Big [\mathbb {A}\mathscr {P}(\mu , z_\mu (\mu ))+\mathbb {F}(\mu ,z_\mu (\mu ))+\int _0^\mu {\mathscr {K}}(\mu , \nu , z(\nu ))d\nu \Big ]d\mu \\- & {} \sum _{0<\nu _k<\nu }\mathbb {B}^{-1}{\mathscr {S}}(\nu _j-\nu _i){\mathcal {I}}_k(z(\nu _j)). \end{aligned}$$

Therefore,

$$\begin{aligned}{} & {} {z(0)+\sum _{j=1}^n c_j z(\nu _j)}\\= \; & {} \mathbb {B}^{-1}{\mathscr {S}}(\nu _j) \mathcal {E}z_0 +\sum _{i=1}^n c_i\mathbb {B}^{-1}\mathcal {S}(\nu _j) \mathscr {E} \Big \{ \mathbb {B}^{-1} \mathscr {P}(\nu , z(\nu ))-\mathbb {B}^{-1} {\mathscr {S}}(\nu _i) \mathcal {E}(0,z(0))\}\\- & {} \sum _{0<\nu _k<\nu }\mathbb {B}^{-1}{\mathscr {S}}(\nu _j-\nu _i){\mathcal {I}}_k(z(\nu _k))\\+ & {} \sum _{i=1}^n c_i \mathbb {B}^{-1}{\mathscr {S}}(\nu _j) \mathcal {E} \Big \{\int _0^{\tau _i}{\mathscr {S}}(\nu _i-\mu )\mathbb {B}^{-1}\Big [\mathbb {A}\mathscr {P}(\mu , z_\mu (\nu ))+\mathbb {F}(\mu , z_\mu (\mu ))\\+ & {} \int _0^\mu {\mathscr {K}}(\mu , \nu , z(\nu )) d\nu \Big ]d\mu \Big \} +\mathbb {B}^{-1}{\mathscr {S}}(\nu _j) \mathscr {P}(0,z(0))-\mathbb {B}^{-1}\mathscr {P}(\nu _j,z_\nu (\nu _j))\\\times & {} \int _0^{\nu _j} {\mathscr {S}}(\nu _j-\mu ) \mathbb {B}^{-1} \Big [\mathbb {A}\mathscr {P}(\mu , z_\mu (\mu ))+\mathbb {F}(\mu ,z_\mu (\mu ))+\int _0^\mu {\mathscr {K}}(\mu , \nu , z(\nu ))d\nu \Big ]d\mu \\- & {} \sum _{0<\nu _k<\nu }\mathbb {B}^{-1}{\mathscr {S}}(\nu _j-\nu _i){\mathcal {I}}_k(z(\nu _k))\ \ =\ \ z_0. \end{aligned}$$

### Theorem 5.2

If assumptions ($A_1$)-($A_7$) hold, then (5.1, 5.2, 5.3) has a fuzzy solution on $\mathbb {J}$.

### Proof

Let $\mathcal F_2$ be the subset of ${\mathscr{P}\mathscr{C}}(\mathbb {J},\mathbb {E}_n)$ defined by

$$\begin{aligned} \mathcal F_2=\{z: z(\nu )\in \mathbb {E}_n, |z(\nu )|\le r, \ \text {for}\ \ t\in \mathbb {J} \}. \end{aligned}$$

We define a mapping $\tilde{\mathscr {F}}_1:\mathcal F_2 \rightarrow \mathcal F_2$ by

$$\begin{aligned} (\tilde{\mathscr {F}}_1 z)(\nu )&=\quad \mathbb {B}^{-1} {\mathscr {S}}(\nu )\mathbb {B}\mathcal {E}z_0 +\sum _{i=1}^n c_i \mathbb {B}^{-1}{\mathscr {S}}(\nu ) \mathbb {B}\mathcal {E}\nonumber \\&\times \quad \Big \{ \mathbb {B}^{-1} \mathscr {P}(\nu , z(\nu )-\mathbb {B}^{-1}{\mathscr {S}}(\nu _i)\mathscr {P}(0,z(0))-\sum _{0<\nu _k<\nu }\mathbb {B}^{-1}{\mathscr {S}}(\nu _k-\nu _i){\mathcal {I}}_k(z(\tau _k))\Big \}\nonumber \\&+\quad \sum _{i=1}^n c_i \mathbb {B}^{-1}{\mathscr {S}}(\nu ) \mathbb {B}\mathcal {E}\Big \{ \int _0^{\nu _i}\mathbb {B}^{-1} {\mathscr {S}}(\nu -\mu )\Big [ \mathbb {A}\mathscr {P}(\mu , z(\mu )+\mathbb {F}(\mu ,z(\mu ))\nonumber )\\&+\quad \int _0^\mu {\mathscr {K}}(\mu , \nu , z(\nu ))d\nu \Big ] d\mu \Big \} +\mathbb {B}^{-1} {\mathscr {S}}(\nu )\mathscr {P}(0,z(0))-\mathbb {B}^{-1} \mathscr {P}(\nu , z(\nu ))\nonumber \\&+ \int _0^\nu {\mathscr {S}}(\nu -\mu )\mathbb {B}^{-1} \Big [ \mathbb {A}\mathscr {P}(\mu , z(\mu ))+ \mathbb {F}(\mu , z(\mu ))+\int _0^\mu {\mathscr {K}}(\mu , \nu , z(\nu ))d\nu \Big ] d\mu \nonumber \\&-\sum _{0<\nu _k<\nu }\mathbb {B}^{-1}{\mathscr {S}}(\nu _k-\nu _i){\mathcal {I}}_k(z(\nu _k)). \end{aligned}$$

To begin, we illustrate that the operator $\tilde{\mathscr {F}}_1$ transfers $\mathcal F_2$ to itself. Now

$$\begin{aligned} |(\tilde{\mathscr {F}}_1 z)(\nu )|= & {} |\mathbb {B}^{-1} {\mathscr {S}}(\nu )\mathbb {B}\mathcal {E}z_0 +\sum _{i=1}^n c_i \mathbb {B}^{-1}{\mathscr {S}}(\nu ) \mathbb {B}\mathcal {E}\nonumber \\{} & {} \times \Big \{ \mathbb {B}^{-1} \mathscr {P}(\nu , z(\nu )-\mathbb {B}^{-1}{\mathscr {S}}(\nu _i)\mathscr {P}(0,z(0))-\sum _{0<\nu _k<\nu }\mathbb {B}^{-1}{\mathscr {S}}(\nu _k-\nu _i){\mathcal {I}}_k(z(\nu _k))\Big \}\nonumber \\{} & {} +\sum _{i=1}^n c_i \mathbb {B}^{-1}{\mathscr {S}}(\nu ) \mathbb {B}\mathcal {E}\Big \{ \int _0^{\nu _i}\mathbb {B}^{-1} {\mathscr {S}}(\nu -\mu )\Big [ \mathbb {A}\mathscr {P}(\mu , z(\mu )+\mathbb {F}(\mu ,z(\mu ))\nonumber )\\{} & {} +\int _0^\mu {\mathscr {K}}(\mu , \nu , x(\tau ))d\tau \Big ] d\mu \Big \} +\mathbb {B}^{-1} {\mathscr {S}}(\nu )\mathscr {P}(0,z(0))-\mathbb {B}^{-1} \mathscr {P}(\nu , z(\nu ))\nonumber \\{} & {} + \int _0^\nu {\mathscr {S}}(\nu -\mu )\mathbb {B}^{-1} \Big [ \mathbb {A}\mathscr {P}(\mu , z\mu ))+ \mathbb {F}(\mu , z(\mu ))+\int _0^\mu {\mathscr {K}}(\mu , \nu , z(\nu ))d\tau \Big ] d\mu \nonumber \\{} & {} -\sum _{0<\nu _k<\nu }\mathbb {B}^{-1}{\mathscr {S}}(\nu _k-\nu _i){\mathcal {I}}_k(z(\nu _k))|\\\le & {} \quad \mathbb {M}_b |\mathbb {B}\mathcal {E}z_0|\mathbb {M}_s+\mathbb {M}_b\mathbb {M}_s{\mathbb {L}}_p+b\mathbb {M}_b\mathbb {M}_s{\mathbb {L}}_pr+b\mathbb {M}_b\mathbb {M}_s[{\mathbb {L}}_pr+b{\mathbb {L}}_kr]\\{} & {} + \mathbb {M}_c \mathbb {M}_b^2|{\mathscr {B}}\mathscr {E}|\mathbb {M}_s\Big [\mathbb {M}_s{\mathbb {L}}_pr+\mathbb {M}_s{\mathbb {L}}_p+b\mathbb {M}_s[\mathscr {L}_fr+a{\mathbb {L}}_k]+\mathbb {M}_s {\mathbb {L}}_i\Big ]+\mathbb {M}_s{\mathbb {L}}_i. \end{aligned}$$

Let $$\mathbb {M}_b |\mathbb {B}\mathcal {E}z_0|\mathbb {M}_s+\mathbb {M}_b\mathbb {M}_s{\mathbb {L}}_p+a\mathbb {M}_b\mathbb {M}_s{\mathbb {L}}_pr+a\mathbb {M}_b\mathbb {M}_s[{\mathbb {L}}_pr+a{\mathbb {L}}_kr] +\mathbb {M}_c \mathbb {M}_b^2|{\mathscr {B}}\mathcal {E}|\mathbb {M}_s\Big [\mathbb {M}_s{\mathbb {L}}_pr+\mathbb {M}_s{\mathbb {L}}_p+a\mathbb {M}_s[\mathscr {L}_fr+a{\mathbb {L}}_k]+\mathbb {M}_s {\mathbb {L}}_i\Big ]+\mathbb {M}_s{\mathbb {L}}_i= \mathfrak {L}_B$$. Hence,

$$\begin{aligned} |(\tilde{\mathscr {F}}_1 z)(\nu )|\le \mathfrak {L}_B. \end{aligned}$$

Therefore $\tilde{\mathscr {F}}_1$ maps $\mathcal {F}_2$ into itself. Moreover, if $z, y \in \mathcal {F}_2,$ then

$$\begin{aligned}{} & {} { \mathscr {H}^d\Big ([\tilde{\mathscr {F}}_1 z)(\nu )]^\lambda , [(\tilde{\mathscr {F}}_1 y)(\nu )]^\lambda \Big )}\\= & {} \mathscr {H}^d\Big ( \Big [\mathbb {B}^{-1} {\mathscr {S}}(\nu )\mathbb {B}\mathcal {E}z_0 +\sum _{i=1}^n c_i \mathbb {B}^{-1}{\mathscr {S}}(\nu ) \mathbb {B}\mathcal {E}\nonumber \\{} & {} \times \quad \Big \{ \mathbb {B}^{-1} \mathscr {P}(\nu , z(\nu )-\mathbb {B}^{-1}{\mathscr {S}}(\nu _i)\mathscr {P}(0,z(0))-\sum _{0<\nu _k<\nu }\mathbb {B}^{-1}{\mathscr {S}}(\nu _k-\nu _i){\mathcal {I}}_k(z(\nu _k))\Big \}\nonumber \\{} & {} +\sum _{i=1}^n c_i \mathbb {B}^{-1}{\mathscr {S}}(\nu ) \mathbb {B}\mathcal {E}\Big \{ \int _0^{\nu _i}\mathbb {B}^{-1} {\mathscr {S}}(\nu -\mu )\Big [ \mathbb {A}\mathscr {P}(\mu , z(\mu )+\mathbb {F}(\mu ,z(\mu ))\nonumber )\\{} & {} +\int _0^\mu {\mathscr {K}}(\mu , \nu , z(\nu ))d\nu \Big ] d\mu \Big \} +\mathbb {B}^{-1} {\mathscr {S}}(\nu )\mathscr {P}(0,z(0))-\mathbb {B}^{-1} \mathscr {P}(\nu , z(\nu ))\nonumber \\{} & {} + \int _0^\nu {\mathscr {S}}(\nu -\mu )\quad \mathbb {B}^{-1} \Big [ \mathbb {A}\mathscr {P}(\mu , z(\mu ))+ \mathscr {F}(\mu , z(\mu ))+\int _0^\mu {\mathscr {K}}(\mu , \nu , z(\nu ))d\nu \Big ] d\mu \nonumber \\{} & {} -\sum _{0<\nu _k<\nu }\mathbb {B}^{-1}{\mathscr {S}}(\nu _k-\nu _i){\mathcal {I}}_k(z(\nu _k))\Big ]^\lambda ,\Big [ \mathbb {B}^{-1} {\mathscr {S}}(\tau )\mathbb {B}\mathbb {E}y_0 +\sum _{i=1}^n c_i \mathbb {B}^{-1}{\mathscr {S}}(\nu ) \mathbb {B}\mathcal {E}\\{} & {} \times \Big \{ \mathbb {B}^{-1} \mathscr {P}(\nu , y(\nu )-\mathbb {B}^{-1}{\mathscr {S}}(\nu _i)\mathscr {P}(0,y(0))-\sum _{0<\nu _k<\nu }\mathbb {B}^{-1}{\mathscr {S}}(\nu _k-\nu _i){\mathcal {I}}_k(y(\nu _k))\Big \}\\{} & {} +\sum _{i=1}^n c_i \mathbb {B}^{-1}{\mathscr {S}}(\nu ) \mathbb {B}\mathcal {E}\Big \{ \int _0^{\nu _i}\mathbb {B}^{-1} {\mathscr {S}}(\nu -\mu )\Big [ \mathbb {A}\mathscr {P}(\mu , y(\mu )+\mathbb {F}(\mu ,y(\mu ))\nonumber )\\{} & {} +\int _0^\mu {\mathscr {K}}(\mu , \nu , y(\nu ))d\nu \Big ] d\mu \Big \} +\mathbb {B}^{-1} {\mathscr {S}}(\nu )\mathscr {P}(0,z(0))-\mathbb {B}^{-1} \mathscr {P}(\tau ,y(\tau ))\nonumber \\{} & {} + \int _0^\nu {\mathscr {S}}(\nu -\mu ){\mathscr {B}}^{-1} \Big [ \mathscr {A}\mathscr {P}(\mu , y(\mu ))+ \mathscr {F}(\mu , y(\mu ))+\int _0^\mu {\mathscr {K}}(\mu , \tau , y(\tau ))d\tau \Big ] d\mu \nonumber \\{} & {} -\sum _{0<\nu _k<\nu }{\mathscr {B}}^{-1}{\mathscr {S}}(\tau _k-\tau _i){\mathcal {I}}_k(y(\tau _k))\Big ]^\lambda \Big )\\\le & {} \Big [ \mathbb {M}_b |{\mathscr {B}}\mathscr {E}z_0|\mathbb {M}_s+\mathbb {M}_b\mathbb {M}_s{\mathbb {L}}_p+b\mathbb {M}_b\mathbb {M}_s{\mathbb {L}}_pr+a\mathbb {M}_b\mathbb {M}_s[{\mathbb {L}}_pr+b{\mathbb {L}}_kr]\\{} & {} + \mathbb {M}_c \mathbb {M}_b^2|{\mathscr {B}}\mathscr {E}|\mathbb {M}_s\Big [\mathbb {M}_s{\mathbb {L}}_pr+\mathbb {M}_s{\mathbb {L}}_p+a\mathbb {M}_s[\mathscr {L}_fr+a{\mathbb {L}}_k]+\mathbb {M}_s {\mathbb {L}}_i\Big ]+\mathbb {M}_s{\mathbb {L}}_i\Big ]\mathscr {H}^d\Big ([x(\tau )]^\lambda ,[y(\tau )]^\lambda \Big )\\\le & {} \mathfrak {L}_B {\mathcal {H}}^d\Big ([z(\nu )]^\lambda ,[y(\nu )]^\lambda \Big ). \end{aligned}$$

Now,

$$\begin{aligned} d^\infty \Big ([\tilde{\mathscr {F}}_1 z(\nu )]^\lambda ,[\tilde{\mathscr {F}}_1 y(\nu )]^\lambda \Big )&\le \quad \sup _{\lambda \in (0,1)}\mathscr {H}^d \Big ([\tilde{\mathscr {F}} z(\nu )]^\lambda ,[\tilde{\mathscr {F}} y(\nu )]^\lambda \Big )\\&\le \quad \mathfrak {L}_Bd^\infty \Big (z(\nu ),y(\nu )\Big ). \end{aligned}$$

Therefore

$$\begin{aligned} \mathscr {H}^1\Big ([\tilde{\mathscr {F}}_1 z(\nu )]^\lambda ,[\tilde{\mathscr {F}}_1 y(\nu )]^\lambda \Big )&\le \quad \sup _{\lambda \in (0,1)}d^\infty \Big ([{\mathscr {F}} z(\nu )]^\lambda ,[{\mathscr {F}} y(\nu )]^\lambda \Big )\\&\le \quad \mathfrak {L}_B \mathscr {H}^1 (z(\nu ),y(\nu )). \end{aligned}$$

Since $\mathfrak {L}_B<1,$ the above equation, the operator $\tilde{\mathscr {F}}_ 1$ is a contraction on $\mathbb {E}_n$, the Banach fixed point theorem indicates that there exists a unique fixed point *z* such that $(\tilde{\mathscr {F}}_ 1 z)(\nu ) = z (\nu ).$ At this fixed point, the problems (5.1)–(5.3) arrive at their conclusion.

## Case study

Consider the fuzzy neutral indegrodifferential equation with non-local condition

6.1 $$\begin{aligned} (x(\tau )-\tilde{\varvec{4}}\tau x^2(\tau ))^\prime= & {} \tilde{\varvec{2}}[x(\tau )+\tau x^2(\tau )]+\tilde{\varvec{3}}\tau x(\tau )^2 \end{aligned}$$

6.2 $$\begin{aligned} x(0)= & {} \sum _{i=1}^n c_i x((\tau _k)) \end{aligned}$$

6.3 $$\begin{aligned} \Delta x(\tau _k)={\mathcal {I}}_k(x(\tau _k))= & {} \varvec{2}e^{2\tau } x(\tau ^-). \end{aligned}$$

Let $$\mathscr {P}(\tau , x(\tau ))=\varvec{4}\tau x^2(\tau ), \mathscr {F}(\tau , x(\tau )=\tilde{\varvec{2}}x^2(\tau ), \int _0^\tau {\mathscr {K}}(\tau ,\mu ,x(\mu ))d\mu =\tilde{\varvec{3}}\tau x^2(\tau )$$, $${\mathcal {I}}_k(x(\tau _k))= \varvec{2}e^{2\tau }x(\tau _k^-).$$

The $\lambda$ level set of fuzzy numbers

$$\begin{aligned}{} & {} \tilde{\varvec{0}}: [0]^\lambda =[\lambda -1,1-\lambda ];\\{} & {} \tilde{\varvec{2}}: [2]^\lambda =[\lambda +1,3-\lambda ];\\{} & {} \tilde{\varvec{3}}: [3]^\lambda =[\lambda +2,4-\lambda ];\\{} & {} \tilde{\varvec{4}}: [4]^\lambda =[\lambda +3,5-\lambda ]; \end{aligned}$$

Now $\alpha$ level set of functions are

$$\begin{aligned} {[}\mathscr {F}(\tau , x(\tau ){]}^\lambda= & {} [\tilde{\varvec{2}}\tau x^2(\tau )]^\lambda \\= & {} \tau \Big [ (\lambda +1) x_l^\lambda (\tau )^2, (2-\lambda )x_r^\lambda (\tau ^2) \Big ]\\ {[}\mathscr {P}(\tau , x(\tau )){]}^\lambda= & {} [\tilde{\varvec{4}}\tau x(\tau )]^\lambda \\= & {} \tau \Big [ (\lambda +3) x_l^\lambda (\tau ), (5-\lambda )x_r^\lambda (\tau ) \Big ]\\ \Big [\int _0^\tau {\mathscr {K}}(\tau ,\mu , x(\mu ))d\mu \Big ]^\lambda= & {} [\tilde{\varvec{3}}\tau x(\tau )^2]^\lambda \\= & {} \tau \Big [ (\lambda +2) x_l^\lambda (\tau )^2, (4-\lambda )x_r^\lambda (\tau )^2 \Big ]. \end{aligned}$$

The $\lambda$- level set of $\sum _{i=1}^n c_i x(\tau _i)$:$\Big [\sum _{i=1}^n c_i x(\tau _i)\Big ]^\lambda =\Big [\sum _{i=1}^n c_i x_l^\lambda (\tau _i),\sum _{i=1}^n c_i x_r^\lambda (\tau _i)\Big ]$

$$\begin{aligned} {\mathcal {H}}^d\Big ([\mathscr {F}(\tau ,x(\tau ))]^\lambda ,[\mathscr {F}(\tau ,y(\tau ))]^\lambda \Big )= & {} {\mathcal {H}}^d\Big (\tau [(\lambda +1)(x_l^\lambda (\tau )), (3-\lambda )(x_r^\lambda (\tau ))],\\{} & {} \tau [(\lambda +1)(y_l^\lambda (\tau )), (3-\lambda )(y_r^\lambda (\tau ))]\Big )\\= & {} \tau \max \{(\lambda +1)|(x_l^\lambda (\tau ))-(y_l^\lambda (\tau ))|,(3-\lambda )|(x_r^\lambda (\tau ))-(y_r^\lambda (\tau ))|\}\\= & {} \tau \max \{(\lambda +1)|x_l^\lambda (\tau )+y_l^\lambda (\tau )||x_l^\lambda (\tau )-y_l^\lambda (\tau )|,\\{} & {} (3-\lambda )|x_r^\lambda (\tau ))+(y_r^\lambda (\tau ))||x_r^\lambda (\tau )-y_r^\lambda (\tau )|\}\\\le & {} (3-\lambda )t|x_r^\lambda (\tau ))+y_r^\lambda (\tau )|\max \{|x_l^\lambda (\tau )-y_r^\lambda (\tau )|,|x_r^\lambda (\tau )-y_r^\lambda (\tau )|\}\\\le & {} (3-\lambda )b|x_r^\lambda (\tau ))+y_r^\lambda (\tau )|\max \{|x_l^\lambda (\tau )-y_l^\lambda (\tau )|,|x_r^\lambda (\tau )-y_r^\lambda (\tau )|\}\\\le & {} 3b|x_r^\lambda (\tau ))+y_r^\lambda (\tau )|\max \{|x_l^\lambda (\tau )-y_l^\lambda (\tau )|,|x_r^\lambda (\tau )-y_r^\lambda (\tau )|\}\\= & {} {\mathbb {L}}_f{\mathcal {H}}^d\Big ([x(\tau )]^\lambda ,[y(\tau )]^\lambda \Big ), \end{aligned}$$

where ${\mathbb {L}}_f=3b|x_r^\lambda (\tau ))+y_r^\lambda (\tau )|$ complies with the inequality indicated within the case of $(A_2)$.

$$\begin{aligned} {\mathcal {H}}^d\Big ([\sum _{i=1}^n c_i x(\tau _i)]^\lambda , [\sum _{i=1}^n c_i y(\tau _i)]^\lambda \Big )= & {} {\mathcal {H}}^d\Big (\Big [\sum _{i=1}^n c_i x_l^\lambda (\tau _i),\sum _{i=1}^n c_i x_r^\lambda (\tau _i)\Big ],\Big [\sum _{i=1}^n c_i y_l^\lambda (\tau _i),\sum _{i=1}^n c_i y_r^\lambda (\tau _i)\Big ]\Big )\\\le & {} \mathbb {M}_c{\mathcal {H}}^d \Big ([x(\tau _i]^\lambda ,y(\tau _i]^\lambda \Big ), \end{aligned}$$

where $\mathbb {M}_c=|\sum _{i=1}^n c_i|$ complies with the inequality indicated within the case of $(A_2)$.

$$\begin{aligned} {\mathcal {H}}^d\Big ([\mathscr {P}(\tau ,x(\tau ))]^\lambda ,[\mathscr {P}(\tau ,y(\tau ))]^\lambda \Big )= & {} {\mathcal {H}}^d\Big (\tau [(\lambda +3)(x_l^\lambda (\tau )^2), (5-\lambda )(x_r^\lambda (\tau )^2)],\\{} & {} \tau [(\lambda +1)(y_l^\alpha (\tau )^2), (3-\lambda )(y_r^\lambda (\tau ))^2]\Big )\\= & {} \tau \max \{(\lambda +1)|(x_l^\lambda (\tau ))-(y_l^\lambda (\tau ))|,(3-\lambda )|(x_r^\lambda (\tau ))-(y_r^\lambda (\tau ))|\}\\\le & {} 5b{\mathcal {H}}^d \Big ([x(\tau _i)]^\lambda ,y(\tau _i)]^\lambda \Big )={\mathbb {L}}_p {\mathcal {H}}^d \Big ([x(\tau _i)]^\lambda ,y(\tau _i)]^\lambda \Big ), \end{aligned}$$

where, ${\mathbb {L}}_p=5b|x_r^\lambda (\tau ))+y_r^\lambda (\tau )|$ fulfills the inequality stipulated in premise $(A_4)$.

$$\begin{aligned} {\mathcal {H}}^d\Big (\Big [\int _0^\tau {\mathscr {K}}(\tau ,\mu , x(\mu ))\Big ]^\lambda ,\Big [\int _0^\tau {\mathscr {K}}(\tau ,\mu , x(\mu ))\Big ]^\lambda \Big )&=\quad {\mathcal {H}}^d\Big (\tau [(\lambda +2)(x_l^\lambda (\tau )^2) , (4-\lambda )(x_r^\lambda (\tau )^2)],\\&\quad \tau [(\lambda +1)(y_l^\lambda (\tau )^2) , (3-\lambda )(y_r^\lambda (\tau ))^2]\Big )\\&=\quad \tau \max \{(\lambda +2)|(x_l^\lambda (\tau ))-(y_l^\lambda (\tau ))|,\\&\quad (4-\lambda )|(x_r^\lambda (\tau ))-(y_r^\lambda (\tau ))|\}\\&\le \quad 4b{\mathcal {H}}^d \Big ([x(\tau _i)]^\lambda ,y(\tau _i)]^\lambda \Big )={\mathbb {L}}_k {\mathcal {H}}^d \Big ([x(\tau _i)]^\lambda ,y(\tau _i)]^\lambda \Big ), \end{aligned}$$

where, ${\mathbb {L}}_k=4b|x_r^\lambda (\tau ))+y_r^\lambda (\tau )|$ meets the inequality stated in the scenario $(A_5)$.

$$\begin{aligned} {\mathcal {H}}^d\Big ([{\mathcal {I}}_k(x_k(\tau ^-))]^\lambda ,[{\mathcal {I}}_k(y_k(\tau ^-))]^\lambda \Big )&=\quad {\mathcal {H}}^d\Big (\Big [ \varvec{2}e^{-2\tau }x(\tau _i)\Big ]^\lambda ,[ \varvec{2}e^{-2\tau }y(\tau ^-)\Big ]^\lambda \Big )\\&\le \quad (3-\lambda ) \varvec{2}e^{-2\tau } {\mathcal {H}}^d ([x(\tau _i)]^\lambda ,y(\tau _i)]^\lambda )\\&\le {\mathbb {L}}_i{\mathcal {H}}^d \Big ([x(\tau _i)]^\lambda ,y(\tau _i)]^\lambda \Big ), \end{aligned}$$

where, ${\mathbb {L}}_i=3e^{-2b}$ satisfies the inequality which is given in condition $(A_7)$. As a result, all of Theorem 5.1’s requirements are met. As an outcome, the fuzzy solution for the system (6.1, 6.2, 6.3) appears unique.

## Conclusion

The outcomes of this work demonstrated the existence of specific types of impulsive neutral integrodifferential equations with Sobolev-type non-local conditions in a fuzzy environment. Fuzzy intervals that are normal, convex, upper semi-continuous, and compactly supported were used in conjunction with the fixed point strategy to examine the findings. An example is given in order to illustrate the concept for each case. Future research will expand this type of study to include control theory and fractional calculus.

## Data Availability

Not Applicable.
